# Serum VIP and IL‐1β Levels Are Associated With Anxiety and Depression in Patients With Parkinson's Disease: A Cross‐Sectional Study

**DOI:** 10.1002/brb3.71742

**Published:** 2026-09-01

**Authors:** Liangyu Li, Xiaoyang Jia, Ying Shi, Zexin Yuan, Caoyuan Niu, Yujing Huang, Xiaoli Zhang, Honglei Li, Tiantian Han, Yanji Cui, Pengge Fei, Guihua Wang, Ailing Du

**Affiliations:** ^1^ Department of Neurology The Second Affiliated Hospital of Henan Medical University Xinxiang China; ^2^ School of Biomedical Engineering Shenzhen University Medical School Shenzhen University Shenzhen Guangdong China

**Keywords:** anxiety, depression, interleukin‐1β (IL‐1β), Parkinson's disease, vasoactive intestinal peptide (VIP)

## Abstract

**Background:**

Anxiety and depressive symptoms frequently accompany Parkinson's disease (PD), yet the biological processes underlying these affective complications and their potential peripheral indicators remain incompletely understood. This study evaluated whether circulating concentrations of vasoactive intestinal peptide (VIP), interleukin‐1β (IL‐1β), and tumor necrosis factor‐alpha (TNF‐α) differed according to the presence of anxiety and depressive symptoms among individuals with PD.

**Methods:**

This exploratory study included 56 individuals with PD and 40 neurologically healthy participants matched for age and sex who were enrolled at the same institution. Clinical characterization included Hoehn–Yahr staging, assessment of daily living function (ADL), cognitive evaluation using MMSE, and measurement of anxiety and depressive symptoms with HAMA‐14 and HAMD‐17. PD‐AD status was determined when participants simultaneously met the predefined cutoff values for both anxiety and depression assessment scales. Circulating concentrations of VIP, TNF‐α, and IL‐1β were measured using enzyme‐linked immunosorbent assays. Relationships between biomarkers and clinical variables were examined through Spearman correlation testing, binary logistic regression modeling, and receiver operating characteristic (ROC) analysis.

**Results:**

Relative to healthy participants, the PD group showed lower circulating VIP concentrations and higher IL‐1β and TNF‐α concentrations, with all comparisons reaching statistical significance (*p* < 0.01). Among participants with PD, those meeting criteria for anxiety and depression showed an additional reduction in VIP accompanied by increased IL‐1β concentrations. Lower VIP concentrations were associated with higher HAMA‐14 and HAMD‐17 scores (*r* = –0.559 and *r* = –0.853, respectively), whereas IL‐1β concentrations demonstrated positive relationships with both symptom scales. ROC analysis identified VIP as the biomarker with the strongest ability to distinguish PD‐AD from PD patients without affective symptoms (AUC = 0.892, 95% CI: 0.799 to 0.986; sensitivity: 0.842; specificity: 0.814). IL‐1β demonstrated a moderate capacity for classification, with an AUC of 0.796 (95% CI: 0.666 to 0.926).

**Conclusion:**

These results suggest that alterations in VIP and IL‐1β are associated with affective symptoms in PD and may contribute to biomarker‐based recognition of PD‐AD in clinical practice. Nevertheless, interpretation of these findings is limited by the observational cross‐sectional design and relatively small cohort size; future studies involving larger longitudinal populations are required for validation.

## 1 Introduction

Parkinson's disease (PD) is a complex neurodegenerative disorder characterized by progressive impairment of dopaminergic pathways and abnormal α‐synuclein aggregation manifested as Lewy body pathology (McGarry et al. 2024). The increasing proportion of older individuals worldwide has contributed to the growing prevalence of PD and has created substantial challenges for healthcare systems. Population‐based studies have estimated that approximately 1.37% of Chinese adults aged 60 years or older are affected by PD, representing nearly four million individuals in this age group (Chen et al. 2020; Qi et al. 2021). Future modeling studies predict that the worldwide PD population could exceed 13 million by 2040, potentially resulting in considerable healthcare and socioeconomic challenges (Feigin et al. 2019).

In addition to classical motor dysfunction, PD involves multiple physiological systems and commonly presents with cognitive, sleep‐related, and emotional disturbances (Bayati et al. 2024; Szatmari et al. 2017; Tsuboi et al. 2022). Importantly, several non‐motor manifestations may appear before obvious motor impairment and are associated with faster clinical deterioration, poorer quality of life, and elevated mortality (Irincu et al. 2022; Toloraia et al. 2022). Therefore, developing objective indicators capable of recognizing and classifying non‐motor subtypes at an early stage represents an important unmet clinical need.

Among the diverse non‐motor manifestations of PD, affective disturbances, particularly anxiety and depressive symptoms, represent clinically important complications that frequently occur together and substantially influence patients’ daily functioning. Previous studies have estimated that anxiety and depression coexist in a considerable proportion of individuals with PD, and these symptoms may emerge during the prodromal phase before prominent motor impairment becomes evident (Choi et al. 2022; Sujith et al. 2024). Evidence from structural and functional studies indicates that abnormalities involving the amygdala, limbic networks, and striatopallidal pathways may participate in the regulation of affective disturbances associated with PD (Buot et al. 2021; Trnka et al. 2018). Multiple pathological processes, including inflammatory activation, redox imbalance, and mitochondrial impairment, have been proposed to contribute to the relationship between PD pathology and affective disturbances (De Oliveira et al. 2023; Liu et al. 2025; Servi et al. 2025; Theofanous and Kourti 2022; Xu et al. 2025). Moreover, peripheral biochemical alterations—such as abnormal levels of uric acid, homocysteine, and lipids—have been proposed as additional contributors to the pathophysiology of PD‐AD. However, the lack of specific and sensitive peripheral biomarkers continues to hinder the early diagnosis and management of affective symptoms in PD.

VIP functions as a neuroactive peptide involved in neuronal regulation and immune modulation in both central and peripheral tissues. Its physiological repertoire includes robust vasodilatory actions, modulation of inflammatory cascades, and the promotion of neuronal survival through neurotrophic support (Fan et al. 2025; Jabłońska et al. 2025; Yonekura et al. 2023). Of particular relevance to PD pathophysiology, VIP exhibits potent anti‐inflammatory and vasodilatory properties; Experimental studies suggest that VIP signaling may regulate microglial responses and reduce excessive production of inflammatory mediators, including TNF‐α and IL‐1β (Langer et al. 2022; Lu et al. 2022; Olson et al. 2016; Reynolds et al. 2010). Given that neuroinflammation is increasingly implicated in the genesis of mood disturbances in PD, we hypothesized that dysregulation of the VIP–cytokine axis may parallel the occurrence of anxiety and depression in this patient population.

Pro‐inflammatory mediators, including TNF‐α and IL‐1β, have been implicated in neuronal injury and dopaminergic system dysfunction observed in PD (Lim et al. 2025). Increased expression of these inflammatory molecules has been detected in peripheral circulation as well as affected brain regions, indicating involvement of both systemic and central immune responses (Bradburn et al. 2019; Qin et al. 2016; Tian et al. 2020). Given the anti‐inflammatory and neuroprotective properties of VIP and the pro‐inflammatory roles of TNF‐α and IL‐1β, an imbalance among these factors may contribute to the occurrence of mood disorders in PD.

Accordingly, we examined circulating VIP, TNF‐α, and IL‐1β concentrations in PD individuals stratified according to the presence or absence of anxiety and depressive symptoms. These findings may improve understanding of the biological basis of affective complications in PD and contribute to future exploration of peripheral indicators for clinical assessment.

## 2 Materials and Methods

### 2.1 Study Subjects

Individuals with a confirmed diagnosis of PD who received neurological care at the Second Affiliated Hospital of Henan Medical University between October 2020 and June 2022 were consecutively enrolled in this exploratory cross‐sectional investigation. Because participants were included consecutively within a fixed recruitment interval, no independent pre‐study sample size estimation was conducted. A total of 56 PD patients were enrolled, including 27 males and 29 females, with a mean age of 62.23 ± 5.37 years. During the same period, 40 healthy individuals undergoing routine physical examinations in the same hospital were enrolled as controls, including 19 males and 21 females, with a mean age of 63.34 ± 5.91 years.

Ethical approval was obtained from the institutional review board, and all enrolled participants provided written informed consent before study procedures. The study procedures complied with the ethical principles outlined in the Declaration of Helsinki and applicable institutional regulations.

### 2.2 Inclusion and Exclusion Criteria

Inclusion criteria for the PD group: PD diagnosis was established according to the Chinese clinical diagnostic criteria for Parkinson's disease; diagnosis confirmed by a board‐certified neurologist with at least an associate senior professional title; ability to independently or with assistance complete the required clinical and psychological assessments; and provision of written informed consent.

Inclusion criteria for the control group: Control participants were frequency‐matched by age and sex and had no clinical manifestations or imaging findings suggestive of parkinsonism.

Exclusion criteria for both groups: parkinsonism or Parkinson‐plus syndromes with identifiable causes; a history of cerebrovascular disease, encephalitis, or poisoning; MMSE < 27 or diagnosis of primary psychiatric disorders; severe dysfunction of the heart, lungs, liver, or kidneys; recent severe infections, malignancies, or treatment with antibiotics or immunosuppressive agents; and pregnancy or breastfeeding.

### 2.3 Data Collection

Demographic and clinical data, including age, sex, education level, comorbidities, and disease duration (for PD patients), were recorded. Education level was classified as low (≤ 6 years) or high (> 6 years). All PD patients underwent brain imaging (CT or MRI) on admission to exclude secondary causes of parkinsonism. Blood biochemical indicators, including homocysteine, blood lipids, uric acid, and high‐sensitivity C‐reactive protein (hs‐CRP), were measured on the following day. Corresponding data from healthy controls were obtained from their physical examinations during the same period. Medication exposure, including antiparkinsonian drugs, antidepressants, anxiolytics, anti‐inflammatory drugs, antibiotics, immunosuppressants, and statins, was reviewed when available; however, complete information on medication dosage and duration was not available for all participants and therefore was not included as an adjusted covariate.

Clinical assessments included disease severity, evaluated using the Hoehn–Yahr (H–Y) scale (Kataoka and Sugie 2021), classifying PD into early (stage I–II), middle (stage III), and late (stage IV–V) stages, and daily living ability assessed by the Activities of Daily Living (ADL) scale (Pashmdarfard and Azad 2020); cognitive function, assessed by the Mini‐Mental State Examination (MMSE), with normal cognition defined as MMSE ≥ 21 for elementary education or below, ≥ 25 for middle school, and ≥ 27 for high school or higher education levels (Folstein et al. 1975; Jia et al. 2021); anxiety, assessed by the 14‐item Hamilton Anxiety Scale (HAMA‐14) (Hamilton 1959); and depression, assessed by the 17‐item Hamilton Depression Rating Scale (HAMD‐17) (Hamilton 1960). Anxiety was defined as HAMA‐14 ≥ 7, and depression was defined as HAMD‐17 ≥ 8. NPD‐AD was defined as both HAMA‐14< 7 and HAMD‐17< 8, whereas PD‐AD was defined as simultaneously meeting the anxiety and depression thresholds. Patients with only anxiety or only depression were excluded from the NPD‐AD versus PD‐AD subgroup comparison to reduce phenotype overlap. Anxiety severity was categorized as none (<7), mild (7–13), moderate (14–20), or severe (≥21), and depression severity was categorized as none (<8), mild (8–16), moderate (17–23), or severe (≥24).

### 2.4 Blood Sample Collection and Measurement of Serum Biomarkers

After overnight fasting, 5‐mL peripheral venous blood specimens were obtained from participants on the morning following admission. Following coagulation at ambient temperature for 30 min, samples underwent centrifugation at 3000 rpm for 15 min to isolate serum. The separated serum was aliquoted into sterile Eppendorf tubes, avoiding repeated freeze–thaw cycles, and stored at –80°C pending analysis.

Serum VIP, TNF‐α, and IL‐1β concentrations were determined using commercially available sandwich‐type ELISA assays according to the manufacturers’ protocols. The assay detection ranges and sensitivities were as follows: VIP (7.82–500 pg/mL; 2.54 pg/mL), TNF‐α (15.63–1000 pg/mL; 6.4 pg/mL), and IL‐1β (15.63–1000 pg/mL; 5.8 pg/mL). Briefly, serially diluted standards and serum specimens were loaded into antibody‐precoated microwell plates, followed by incubation with biotin‐conjugated detection antibodies and subsequent addition of avidin–HRP conjugates. Following a washing step, the chromogenic substrate TMB was introduced; the reaction was terminated with stop solution, and absorbance was measured at 450 nm (±10 nm). Standard curves were constructed for each assay, and sample concentrations were extrapolated from the corresponding optical density values. All measurements were performed in duplicate, with intra‐assay and inter‐assay coefficients of variation reported by the manufacturer of less than 8% and 10%, respectively.

### 2.5 Statistical Analysis

Statistical analyses were conducted with SPSS software (version 25.0; IBM Corp., Armonk, NY, USA). The distribution characteristics of continuous variables were examined using the Shapiro–Wilk test. Variables conforming to a normal distribution are presented as mean ± standard deviation (SD) and were analyzed using Student's *t*‐test or one‐way analysis of variance (ANOVA), whereas variables showing non‐normal distributions are summarized as median values with interquartile ranges (IQR) and assessed using the Mann–Whitney U test or Kruskal–Wallis H test. Differences in categorical variables were examined using the chi‐square test. Associations between biomarker concentrations and clinical measurements were explored using Spearman's rank correlation analysis. Binary logistic regression models were applied to determine variables independently associated with PD and PD‐AD classification. The diagnostic performance of biomarkers was evaluated by receiver operating characteristic (ROC) curve analysis. A two‐sided *p*‐value below 0.05 was considered statistically significant.

## 3 Results

### 3.1 Baseline Characteristics and Scale Scores Between the Control and PD Groups

Demographic characteristics, vascular risk factors, education level, and baseline biochemical indicators, including homocysteine, total cholesterol, hs‐CRP, and uric acid, were comparable between individuals with PD and healthy participants (Tables S1 and S2).

The median MMSE score did not differ significantly between the two groups [26.00 (25.00–27.50) vs. 26.00 (24.00–27.00), *p >* 0.05]. However, both HAMA and HAMD scores were significantly higher in the PD group than in controls (*p <* 0.001). Median HAMA scores were 2.00 (1.00–3.00) in the control group and 9.50 (6.50–15.00) in the PD group; median HAMD scores were 1.00 (0.00–2.00) and 13.00 (6.00–18.00), respectively. Among PD patients, the median ADL score was 72.50 (55.00–85.00), and the median Hoehn–Yahr stage was 2.00 (1.00–3.00) (Table 1).

**TABLE 1:** Comparison of scale scores between the control group and PD group.

Item	Control group (*n* = 40)	PD group (*n* = 56)	*Z*	*p* value
MMSE scores	26.00 (25.00, 27.50)	26.00 (24.00, 27.00)	−0.781^b^	0.435
HAMA scores	2.00 (1.00, 3.00)	9.50 (6.50, 15.00)	−7.841^b^	<0.001
HAMD scores	1.00 (0.00, 2.00)	13.00 (6.00, 18.00)	−7.872^b^	<0.001
ADL scores	—	72.50 (55.00, 85.00)	—	—
H‐Y staging	—	2.00 (1.00, 3.00)	—	—

^b^Mann–Whitney *U* test.

*Note*: Exact *p* values are shown; *p* < 0.05 was considered statistically significant.

### 3.2 Alterations in Circulating VIP, TNF‐α, and IL‐1β Concentrations in PD

**FIGURE 1:**
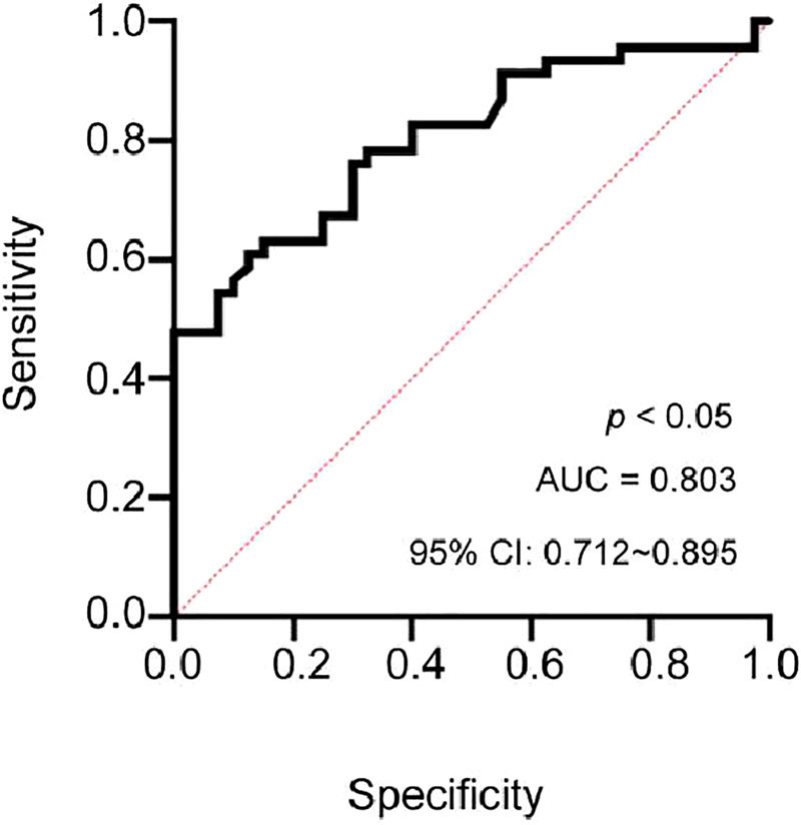
ROC curve of serum TNF‐α for discriminating PD patients from healthy controls.

As shown in Table 2, serum VIP levels were significantly lower in PD patients than in controls (160.37 ± 47.63 vs. 219.86 ± 56.82 pg/mL, *t* = 5.282, *p* < 0.001). Conversely, PD participants exhibited elevated circulating TNF‐α and IL‐1β concentrations compared with controls (TNF‐α: 371.09 ± 62.84 vs. 293.01 ± 41.27 pg/mL; IL‐1β: 287.81 ± 116.73 vs. 217.10 ± 83.91 pg/mL) (Table 2). Exploratory multivariate logistic regression was performed to assess the associations between serum cytokine levels and PD status. The model fit was acceptable (Hosmer–Lemeshow test: *p* = 0.389). The regression equation was: Logit(*P*) = −3.283 − 0.011 (VIP) + 0.015 (TNF‐α) + 0.002 (IL‐1β), with an overall classification accuracy of 73.3%. TNF‐α was independently associated with PD status (*p* = 0.020, OR = 1.015, 95% CI: 1.002 to 1.029) (Table 3). ROC curve analysis demonstrated that serum TNF‐α showed discriminative ability for PD, with an AUC of 0.803 (95% CI: 0.712 to 0.895, *p* < 0.05), sensitivity of 0.630, and specificity of 0.825 at the optimal cutoff value of 334.14 pg/mL (Figure 1).

**TABLE 2:** Alterations in circulating VIP, TNF‐α, and IL‐1β concentrations in PD.

Item	Control group (*n* = 40)	PD group (*n* = 56)	Stat.	*p* value
VIP (pg/mL)	219.86±56.82	160.37±47.63	5.282^a^	<0.001
TNF‐α (pg/mL)	293.01±41.27	371.09±62.84	−4.836^a^	<0.001
IL‐1β (pg/mL)	217.10±83.91	287.81±116.73	−3.181^a^	0.002

^a^ *t*‐test.

*Note*: Exact *p* values are shown. *p* < 0.05 was considered statistically significant.

**TABLE 3:** Logistic regression analysis of serum VIP, TNF‐α, and IL‐1β associated with PD status.

Item	*B*	SE	$\bar{\chi }$ Wald	*p* value	OR	95% CI
X_1_ (VIP)	−0.011	0.006	3.232	0.072	0.989	0.976 to 1.000
X_2_ (TNF‐α)	0.015	0.007	5.378	0.020	1.015	1.002 to 1.029
X_3_ (IL‐1β)	0.002	0.003	0.701	0.403	1.002	0.997 to 1.008
Constant	−3.283	—	—	—	—	—

OR, odds ratio; CI, confidence interval.

*Note: p* < 0.05 was considered statistically significant.

### 3.3 Comparison of Serum Parameters Between PD and PD‐AD Patients

To further investigate the relationship between serum inflammatory markers and emotional symptoms in PD, patients were stratified according to the predefined HAMA‐14 and HAMD‐17 thresholds into two subgroups: PD without anxiety or depression (NPD‐AD, *n* = 19) and PD with comorbid anxiety and depression (PD‐AD, *n* = 27). Patients with only anxiety (*n* = 6) or only depression (*n* = 4) were excluded from this subgroup comparison to reduce phenotype heterogeneity. Diagnoses were jointly confirmed by two attending neurologists.

**FIGURE 2:**
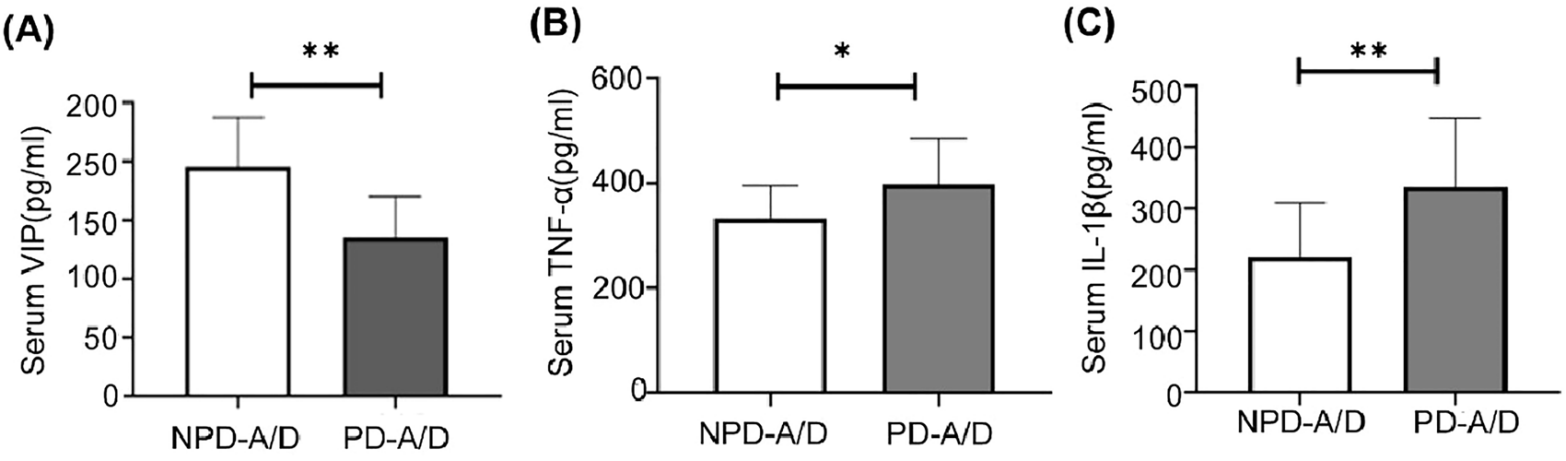
Expression levels of serum VIP, TNF‐α, and IL‐1β in the NPD‐AD group and PD‐AD group. Serum VIP (A), TNF‐α (B), and IL‐1β (C) levels are shown.

Baseline demographic and biochemical parameters did not differ significantly between the NPD‐AD and PD‐AD groups (*p* > 0.05). However, serum biomarker levels showed significant between‐group differences. Mean serum VIP levels were markedly lower in the PD‐AD group than in the NPD‐AD group (135.63 ± 34.51 vs. 195.22 ± 41.53 pg/mL, *t* = 5.322, *p* < 0.001). In contrast, both TNF‐α (391.60 ± 86.55 vs. 337.56 ± 61.61 pg/mL, *t* = 2.767, *p* = 0.008) and IL‐1β (332.92 ± 112.05 vs. 223.91 ± 88.87 pg/mL, *t* = 3.688, *p* < 0.001) were significantly higher in PD‐AD patients (Table 4; Figure 2A–C).

**TABLE 4:** Comparison of serum parameters between the NPD‐AD group and PD‐AD group.

Item	NPD‐AD (*n* = 19)	PD‐AD (*n* = 27)	Stat.	*p* value
VIP (pg/mL)	195.22 ± 41.53	135.63 ± 34.51	5.322^a^	<0.001
TNF‐α (pg/mL)	337.56 ± 61.61	391.60 ± 86.55	2.767^a^	0.008
IL‐1β (pg/mL)	223.91 ± 88.87	332.92 ± 112.05	3.688^a^	<0.001

^a^ *t*‐test.

*Note*: Exact *p* values are shown; *p* < 0.05 was considered statistically significant.

### 3.4 Correlation Analysis of Serum VIP, TNF‐α, and IL‐1β Levels With Disease Progression in PD

**FIGURE 3:**
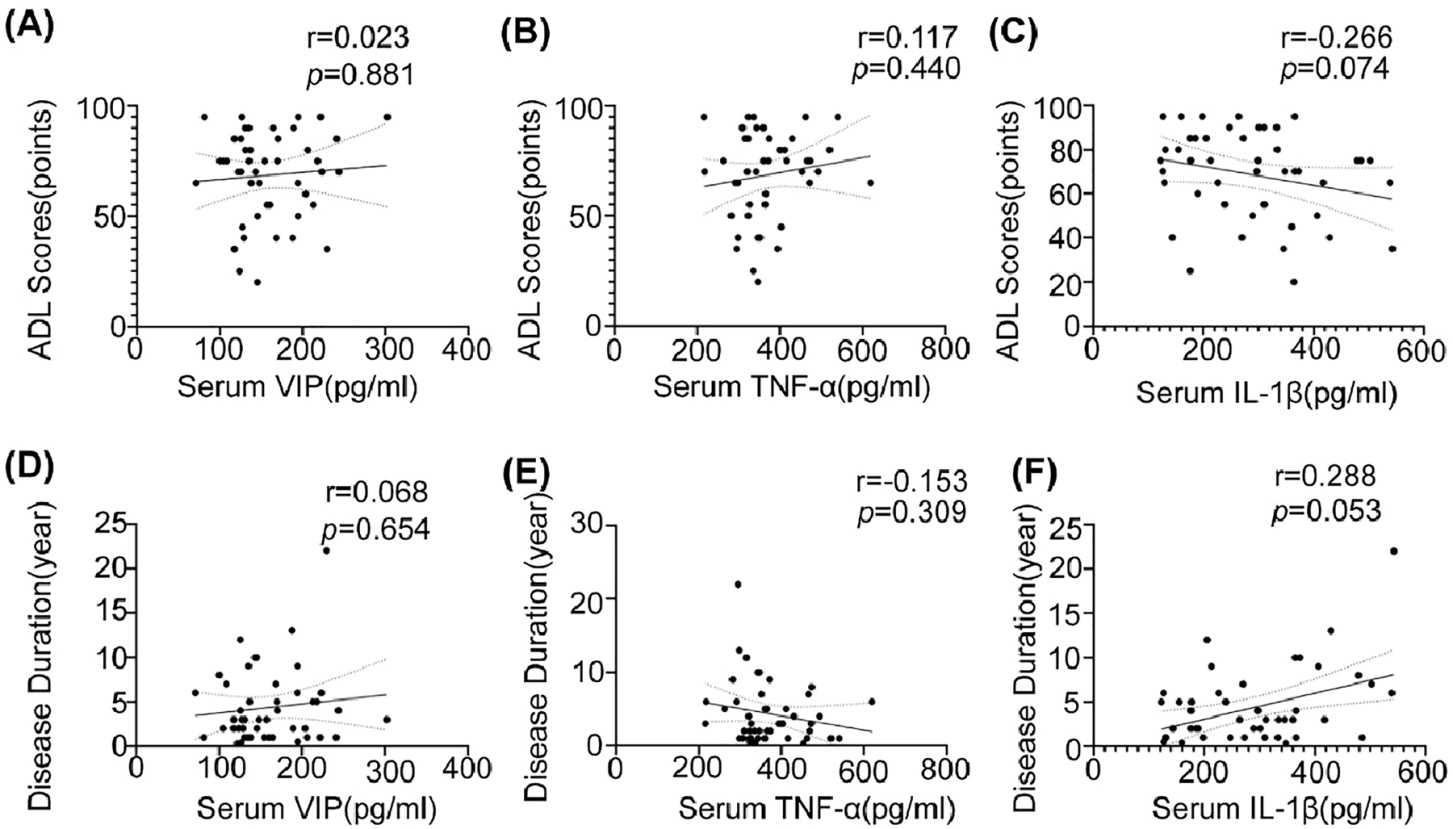
Correlations of ADL scores with serum VIP levels (A), TNF‐α levels (B), and IL‐1β levels (C). Correlations of disease duration with serum VIP levels (D), TNF‐α levels (E), and IL‐1β levels (F) in PD patients.

Correlation analysis indicated that serum VIP, TNF‐α, and IL‐1β levels were not significantly associated with ADL scores (*r* = 0.023, *p =* 0.881; *r* = 0.117, *p =* 0.440; and *r* = –0.266, *p =* 0.074, respectively; *n* = 56) (Figure 3A–C). Similarly, no significant correlations were observed between disease duration and serum levels of VIP (*r* = 0.068, *p =* 0.654), TNF‐α (*r* = –0.153, *p =* 0.309), or IL‐1β (*r* = 0.288, *p =* 0.053) (Figure 3D–F).

**FIGURE 4:**
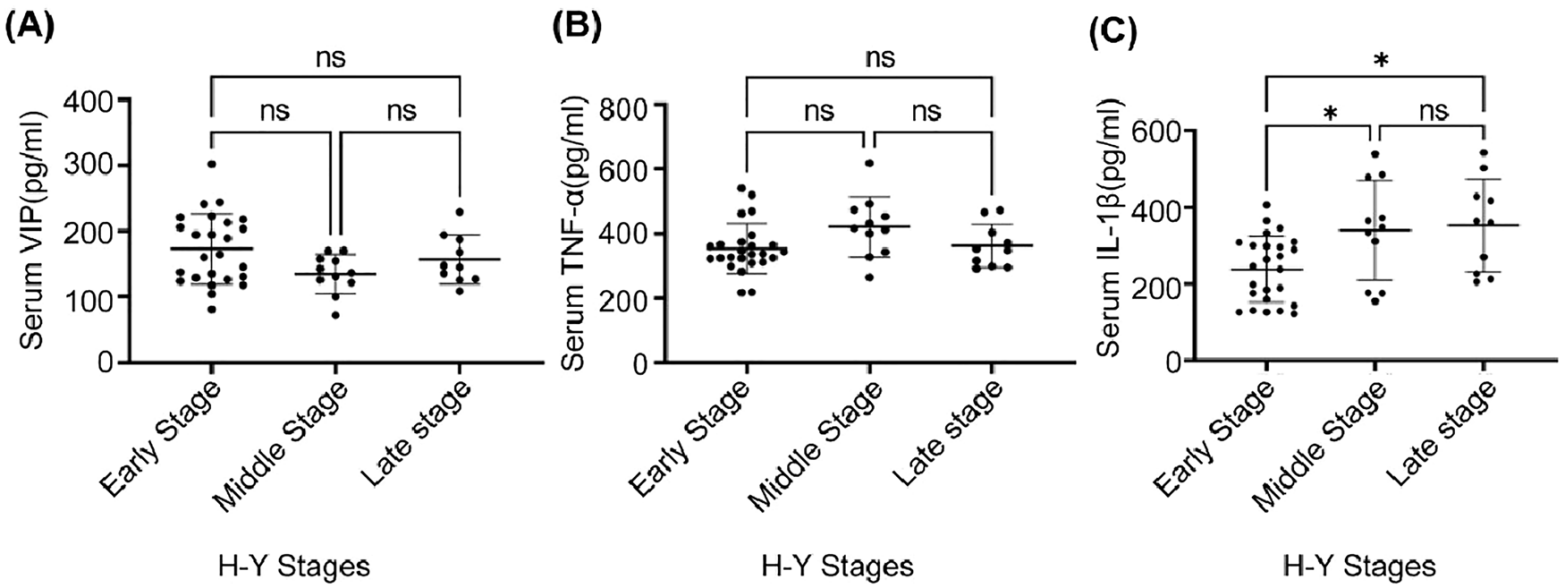
Comparison of serum VIP (A), TNF‐α (B), and IL‐1β (C) levels across different H–Y stages.

To further examine the relationship between biomarker expression and disease stage, PD patients were classified into early (Hoehn–Yahr I–II, *n* = 28), middle (stage III, *n* = 15), and late (stages IV–V, *n* = 13) subgroups. The Kruskal–Wallis H test revealed no significant differences in serum VIP or TNF‐α levels among the three disease stages. However, serum IL‐1β levels were significantly higher in the middle and late stages compared with the early stage (*p =* 0.008), suggesting that IL‐1β elevation may be associated with disease progression (Table 5 and Figure 4A–C).

**TABLE 5:** Comparison of serum VIP, TNF‐α, and IL‐1β Levels across different H‐Y stages.

Item	VIP (pg/mL)	TNF‐α (pg/mL)	IL‐1β (pg/mL)
Early stage (*n* = 28)	164.42 (127.89, 215.62)	337.14 (316.26, 369.73)	246.62 (151.67, 304.45)
Middle stage (*n* = 15)	136.77 (121.83, 157.55)	415.56 (342.78, 473.66)	347.87 (177.33, 478.45)
Late stage (*n* = 13)	146.44 (126.72, 189.24)	340.03 (297.33, 418.67)	362.22 (223.15, 447.14)
H	3.480	5.015	9.719
*p* value	0.175	0.081	0.008

*Note: p* < 0.05 was considered statistically significant.

### 3.5 Correlation of Serum VIP, TNF‐α, and IL‐1β Levels With Anxiety in PD and PD‐AD Patients

**FIGURE 5:**
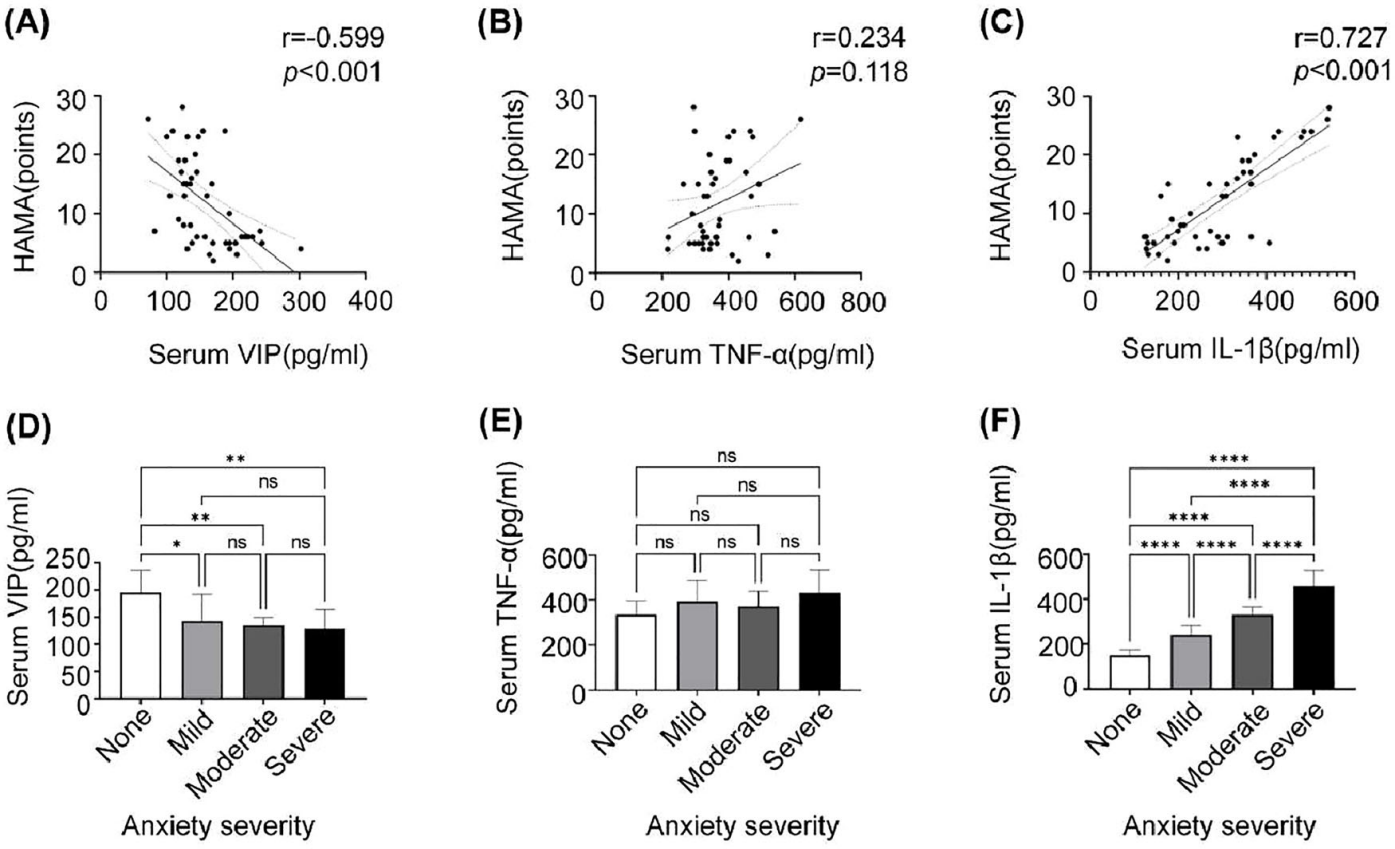
Correlations of serum VIP, TNF‐α, and IL‐1β levels with anxiety symptoms. Correlations of HAMA scores with serum VIP (A), TNF‐α (B), and IL‐1β (C). Distribution of serum VIP levels (D), TNF‐α levels (E), and IL‐1β levels (F) across different anxiety severity groups.

Correlation analysis between serum biomarkers and anxiety severity demonstrated that serum VIP was negatively correlated with HAMA scores (*r* = −0.559, *p* < 0.001, *n* = 46) (Figure 5A), whereas serum IL‐1β was positively correlated with HAMA scores (*r* = 0.727, *p* < 0.001, *n* = 46) (Figure 5C). No significant correlation was observed between serum TNF‐α and HAMA scores (*r* = 0.234, *p* = 0.118, *n* = 46) (Figure 5B).

According to HAMA‐14 assessment, patients were further categorized into no anxiety (*n* = 19), mild anxiety (*n* = 9), moderate anxiety (*n* = 10), and severe anxiety (*n* = 8) groups. The Kruskal–Wallis H test showed that serum VIP levels were significantly lower in anxious PD patients compared with non‐anxious patients (*p* < 0.05), although no significant differences were found among the mild, moderate, and severe anxiety subgroups (Figure 5D and Table 6). Serum TNF‐α levels did not differ significantly among the four groups (*p* > 0.05) (Figure 5E). In contrast, serum IL‐1β levels increased progressively with anxiety severity, showing significant differences across groups (*p* < 0.001) (Figure 5F). These findings indicate that decreased serum VIP and elevated IL‐1β levels are associated with the presence and severity of anxiety symptoms in PD.

**TABLE 6:** Comparison of serum IL‐1β, TNF‐α, and VIP levels across different anxiety severity groups.

Item	VIP (pg/mL)	TNF‐α (pg/mL)	IL‐1β (pg/mL)
None (*n* = 19)	194.91 (164.43, 221.72)	326.83 (310.78, 364.45)	151.19 (132.33, 169.23)
Mild (*n* = 9)	126.44 (111.08, 177.45)	371.42 (319.56, 494.44)	241.72 (201.89, 292.32)
Moderate (*n* = 10)	132.17 (124.67, 143.55)	356.25 (334.14, 415.45)	330.89 (303.56, 365.34)
Severe (*n* = 8)	127.44 (102.32, 152.78)	440.67 (324.09, 473.33)	459.24 (392.56, 523.84)
H	20.453	1.519	29.745
*p* value	<0.001	0.224	<0.001

*Note: p* < 0.05 was considered statistically significant.

### 3.6 Correlation and ROC‐Based Discriminative Analysis of Serum VIP, TNF‐α, and IL‐1β in PD‐AD Patients

**FIGURE 6:**
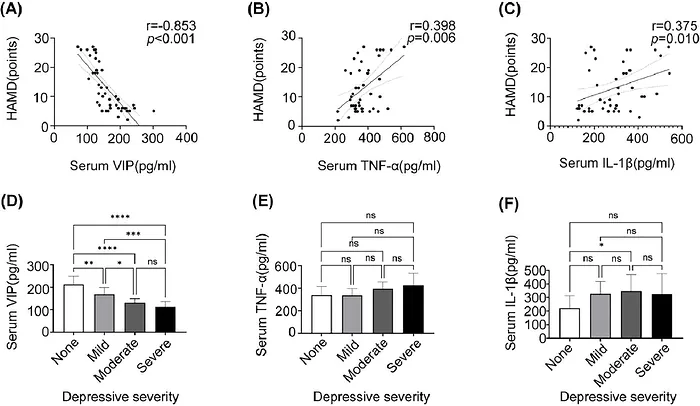
Correlations of serum VIP, TNF‐α, and IL‐1β levels with depressive symptoms. Correlations of HAMD scores with serum VIP (A), TNF‐α (B), and IL‐1β (C). Distribution of serum VIP levels (D), TNF‐α levels (E), and IL‐1β levels (F) across different depression severity groups.

**FIGURE 7:**
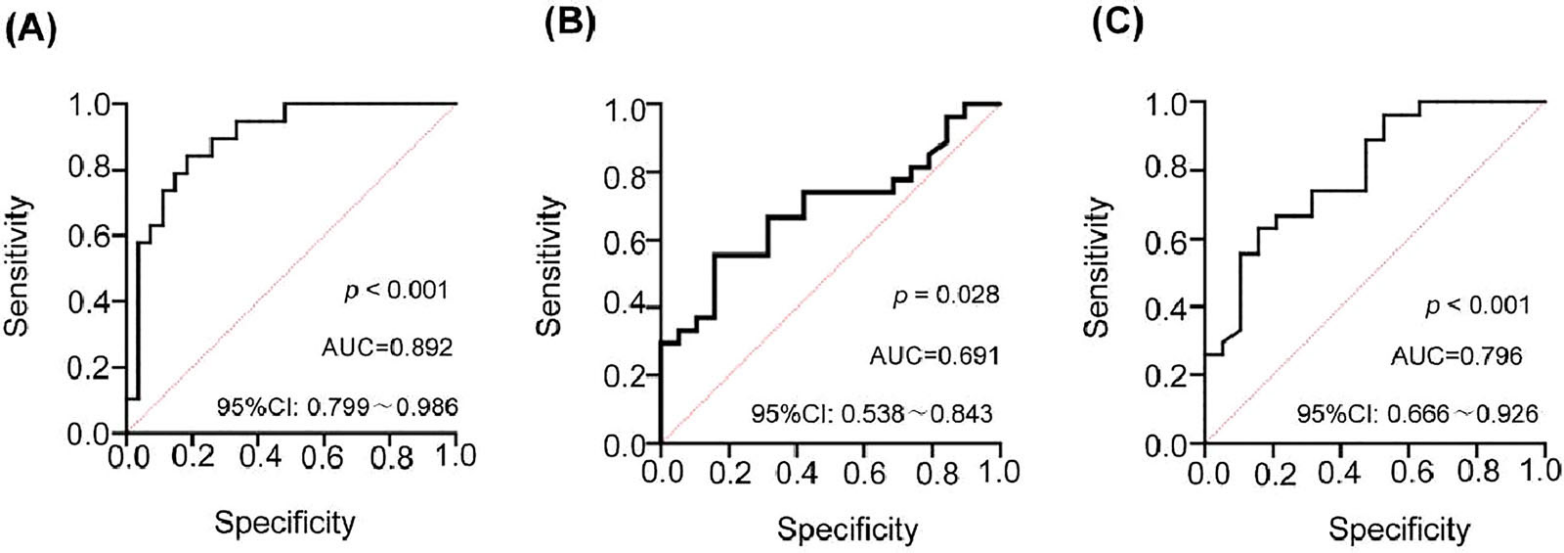
ROC analysis of serum VIP (A), TNF‐α (B), and IL‐1β (C) levels for discriminating PD‐AD patients from NPD‐AD patients.

Correlation analysis demonstrated a strong negative association between serum VIP levels and HAMD scores (*r* = −0.853, *p* < 0.001, *n* = 46), indicating that lower VIP concentrations were linked to more severe depressive symptoms. In contrast, serum TNF‐α and IL‐1β levels showed positive correlations with HAMD scores (*r* = 0.398, *p* = 0.006 and *r* = 0.375, *p* = 0.010, respectively; *n* = 46) (Figure 6A–C), suggesting that elevated inflammatory cytokines were associated with increased depression severity. According to HAMD‐17 classification, patients were divided into the no depression group (*n* = 19), mild depression group (*n* = 11), moderate depression group (*n* = 9), and severe depression group (*n* = 7). The Kruskal–Wallis H test revealed significant group differences in serum VIP levels (*p* < 0.001), with a progressive decline in VIP concentration corresponding to greater depression severity (Table 7 and Figure 6D). Serum TNF‐α levels did not differ significantly among the four groups (*p* > 0.05) (Figure 6E). However, serum IL‐1β levels were significantly higher in patients with mild to severe depression compared with non‐depressed PD patients (*p* = 0.010) (Figure 6F). These findings indicate that reduced serum VIP and elevated IL‐1β are associated with depression in PD. Exploratory binary logistic regression was subsequently conducted to examine biomarker associations with PD‐AD. The regression model yielded the following equation: Logit(*P*) = 7.441 − 0.071 (VIP) + 0.002 (TNF‐α) + 0.013 (IL‐1β), with an overall classification accuracy of 89.1% (Table 8). Serum VIP (*p* = 0.002, OR = 0.932, 95% CI: 0.891 to 0.974) and IL‐1β (*p* = 0.036, OR = 1.014, 95% CI: 1.001 to 1.026) were independently associated with PD‐AD, whereas TNF‐α showed no significant association (*p* = 0.836). ROC curve analyses further evaluated the discriminative performance of these biomarkers. Serum VIP demonstrated the highest discriminative performance for PD‐AD, with an AUC of 0.892 (95% CI: 0.799 to 0.986, *p* < 0.001), sensitivity of 0.842, and specificity of 0.814 at the optimal cutoff value of 156.04 pg/mL (Figure 7A). Serum IL‐1β also showed acceptable discriminative ability, with an AUC of 0.796 (95% CI: 0.666 to 0.926, *p* < 0.001), sensitivity of 0.667, and specificity of 0.789 at the cutoff of 298.14 pg/mL (Figure 7C). In contrast, the discriminative performance of TNF‐α was relatively limited, with an AUC of 0.691 (95% CI: 0.538 to 0.843, *p* = 0.028), sensitivity of 0.556, and specificity of 0.842 at the cutoff of 368.56 pg/mL (Figure 7B).

**TABLE 7:** Comparison of serum IL‐1β, TNF‐α, and VIP levels across different depression severity groups.

Item	VIP (pg/mL)	TNF‐α (pg/mL)	IL‐1β (pg/mL)
None (*n* = 19)	218.22 (187.33, 271.54)	334.13 (287.28, 379.56)	189.89 (131.44, 296.89)
Mild (*n* = 11)	168.56 (139.67, 203.43)	373.03 (303.45, 438.34)	308.44 (246.72, 417.24)
Moderate (*n* = 9)	131.34 (114.67, 153.62)	381.32 (312.84, 463.48)	345.56 (257.63, 433.27)
Severe (*n* = 7)	113.67 (94.22, 127.64)	429.31 (311.44, 492.12)	347.89 (185.33, 478.48)
H	16.496	4.789	11.313
*p* value	<0.001	0.121	0.010

*Note: p* < 0.05 was considered statistically significant.

**TABLE 8:** Logistic regression analysis of serum VIP, TNF‐α, and IL‐1β associated with PD‐AD status.

Item	*B*	SE	$\bar{\chi }$ Wald	*p* value	OR	95% CI
X_1_ (VIP)	−0.071	0.023	9.716	0.002	0.932	0.891 to 0.974
X_2_ (TNF‐α)	0.002	0.009	0.043	0.836	1.002	0.985 to 1.019
X_3_ (IL‐1β)	0.013	0.006	4.405	0.036	1.014	1.001 to 1.026
Constant	7.441	—	—	—	—	—

OR, odds ratio; CI, confidence interval.

*Note: p* < 0.05 was considered statistically significant.

Taken together, these results suggest that decreased serum VIP and increased IL‐1β levels are associated with the occurrence of anxiety and depression in PD patients. Furthermore, serum VIP showed the highest ROC‐based discriminative performance among the three biomarkers for distinguishing PD‐AD from NPD‐AD, although this finding requires validation in independent cohorts.

## 4 Discussion

In the present investigation, we examined circulating VIP, TNF‐α, and IL‐1β concentrations in individuals with PD and further evaluated their relationships with anxiety and depressive symptoms. Compared with healthy participants, the PD group displayed reduced circulating VIP concentrations accompanied by increased TNF‐α and IL‐1β concentrations. Within the PD cohort, individuals presenting with anxiety and depression showed additional decreases in VIP and elevations in IL‐1β compared with those without affective manifestations. Analysis of biomarker–symptom relationships revealed an inverse association between VIP concentrations and affective symptom scores, while IL‐1β concentrations increased in parallel with anxiety and depressive severity. Logistic regression and ROC analyses suggested that VIP and IL‐1β were associated with PD‐AD and had discriminative value in this cohort, with VIP showing the highest AUC. Collectively, our data imply that disturbances in the neurochemical equilibrium between VIP and pro‐inflammatory cytokines are linked to the presence of anxiety and depression in PD. Nevertheless, the cross‐sectional nature of this study precludes causal inferences regarding whether these biomarker alterations precede or result from the development of affective symptoms.

At baseline, there were no significant differences between the PD and control groups in serum levels of homocysteine (HCY), total cholesterol (TC), or uric acid (UA). The absence of significant intergroup differences in homocysteine or lipid profiles contrasts with select prior investigations. Such discrepancies may stem from methodological variations, including our modest sample size, the heterogeneity of patient recruitment criteria, or the confounding influence of long‐term dopaminergic medication regimens. Although several experimental studies have shown that exogenous HCY exacerbates neurodegeneration and oxidative stress in PD models (Custodero et al. 2022; Sharma et al. 2011), clinical evidence remains conflicting. Elevated HCY levels in PD patients are often secondary to long‐term levodopa therapy rather than an independent etiological factor (Müller et al. 2011; Romagnolo et al. 2019; Toth et al. 2010). Similarly, cholesterol metabolism in the brain is complex: while TC contributes to neuronal membrane synthesis and steroid hormone production, excess cholesterol metabolites such as 24S‐hydroxycholesterol may promote oxidative stress and neuronal apoptosis (Dai et al. 2021). Some cohort studies suggest that high TC may lower PD risk (Jiang et al. 2020), whereas others report increased risk in younger individuals with hypercholesterolemia (Fu et al. 2020). These discrepancies likely result from age‐related physiological changes and methodological variations.

Vasoactive intestinal peptide (VIP) has emerged as a key neuroimmune regulatory molecule involved in maintaining neuronal stability and immune balance across central and peripheral systems (Zawilska et al. 2005). The biological actions of VIP are mediated primarily through VPAC1‐ and VPAC2‐dependent signaling pathways, which influence neuronal survival and inflammatory regulation. Studies using PD‐related experimental models have indicated that modulation of VIP activity may counteract dopaminergic neuronal impairment, reduce excessive microglial responses, and suppress inflammatory mediator production, including TNF‐α and IL‐1β (Goff and Goldberg 2019; Zhang et al. 2022). In clinical populations, decreased peripheral VIP concentrations have been associated with greater non‐motor symptom burden, especially affective disturbances assessed by NMSS (Hu et al. 2021). Consistent with these findings, our study showed that serum VIP was lower in PD patients, especially those with comorbid anxiety and depression, and was inversely correlated with HAMA and HAMD scores. These observations suggest that reduced VIP levels may be related to PD‐associated emotional dysfunction, although the direction and mechanism of this association require further investigation.

Our results further revealed that serum TNF‐α and IL‐1β levels were significantly higher in PD patients than in healthy controls, in line with previous reports (Buot et al. 2021; Marogianni et al. 2020). Both cytokines are pivotal mediators of neuroinflammation and may contribute to dopaminergic neuronal injury. TNF‐α promotes microglial activation and amplifies inflammatory signaling via TNFR1, triggering caspase cascades and apoptosis (Buot et al. 2021). It also induces endothelial adhesion molecules, facilitating leukocyte infiltration and sustained neuroinflammatory responses (Fricke‐Galindo et al. 2022). Elevated serum TNF‐α levels have been associated with disease severity, higher HAMD scores, and advanced PD stages (Karpenko et al. 2018; Xiromerisiou et al. 2022). In our cohort, TNF‐α was positively correlated with depressive but not anxious symptoms, suggesting that different inflammatory markers may relate to distinct affective domains. However, its limited ROC‐based discriminative performance indicates that TNF‐α alone is insufficient as an indicator of PD‐AD.

IL‐1β, another key pro‐inflammatory cytokine, contributes to neuronal damage by promoting oxidative stress, amplifying cytokine cascades, and inhibiting neurotrophic factor synthesis (Schulz et al. 2021). Elevated IL‐1β levels have been reported in PD and are closely associated with disease progression and non‐motor symptoms (Wang et al. 2016). Consistent with these findings, our study demonstrated that IL‐1β was significantly increased in PD and PD‐AD patients and correlated positively with both anxiety and depression severity. In the exploratory logistic model, IL‐1β remained associated with PD‐AD together with VIP, supporting the possibility that neuropeptide and inflammatory indicators may provide complementary information. This interpretation should be regarded as preliminary because the model was not externally validated and was not fully adjusted for all clinical covariates.

Collectively, our findings indicate that serum VIP and IL‐1β are associated with affective symptoms in PD and may be useful peripheral indicators for identifying patients with concurrent anxiety and depression. The negative correlation between VIP and mood symptom severity, together with its relatively high AUC in this cohort, suggests potential clinical relevance for screening. However, these results should not be interpreted as establishing causality or prospective clinical performance. Future studies should verify these findings in larger, multicenter, longitudinal cohorts and evaluate whether VIP‐ and IL‐1β‐related pathways have mechanistic or therapeutic relevance.

Several limitations should be acknowledged. First, this was a single‐center cross‐sectional study with a relatively small sample size, especially for the PD‐AD subgroup, and no formal a priori sample size calculation or statistical power analysis was performed. Therefore, the findings should be considered exploratory and may not be generalizable to broader PD populations. Second, potential confounding factors such as antiparkinsonian medications, antidepressants, anxiolytics, anti‐inflammatory drugs, statins, disease duration, and comorbid conditions were not fully adjusted for, which may have influenced serum VIP and cytokine levels. Third, the logistic regression models were primarily biomarker‐based because the limited sample size and incomplete medication data restricted the inclusion of additional clinical covariates; consequently, residual confounding cannot be excluded. Fourth, the ROC results were not validated in an independent external cohort or by internal resampling methods; therefore, the discriminative performance may be overestimated. Fifth, mechanistic validation at the molecular level was not performed. Future multicenter longitudinal studies with larger sample sizes, standardized medication assessment, adjusted models, and validation cohorts are warranted to confirm the clinical relevance of VIP and IL‐1β in PD‐AD.

## 5 Conclusion

In summary, serum VIP levels were reduced and IL‐1β levels were elevated in Parkinson's disease patients with comorbid anxiety and depression. Both biomarkers were associated with affective symptom burden, and VIP showed the highest ROC‐based discriminative performance in this exploratory cohort. These findings suggest that combined assessment of serum VIP and IL‐1β may provide supportive information for identifying mood symptoms in Parkinson's disease, but larger longitudinal and validation studies are required before clinical application can be recommended.

## Author Contributions

**Xiaoli Zhang**: validation, visualization. **Honglei Li**: supervision, data curation. **Xiaoyang Jia**: resources, data curation, conceptualization, methodology, software, formal analysis, validation, investigation, funding acquisition, visualization, project administration, supervision, writing – original draft. **Liangyu Li**: writing – original draft. **Guihua Wang**: funding acquisition. **Caoyuan Niu**: writing – review and editing, writing – original draft. **Yujing Huang**: formal analysis, supervision. **Zexin Yuan**: investigation. **Pengge Fei**: visualization. **Ying Shi**: software. **Tiantian Han**: visualization, project administration. **Yanji Cui**: visualization, supervision. **Ailing Du**: supervision, funding acquisition.

## Funding

This work was supported by the General Project of the Open Program in Psychiatry and Neurology of the Second Affiliated Hospital of Xinxiang Medical University, titled “Expression levels and clinical significance of plasma exosomal miRNAs in patients with Parkinson's disease” (No. XYEFYJSSJ‐2024‐09; awarded to Guihua Wang).

## Ethics Statement

This study was approved by the Ethics Committee of the Second Affiliated Hospital of Henan Medical University (Approval No: XYEFYLL—2024‐80). Informed consent was obtained from all participants. Data are used for this research only.

## Conflicts of Interest

The authors declare that the research was conducted in the absence of any commercial or financial relationships that could be construed as a potential conflict of interest.

## Supporting information




**Table S1**: Comparison of baseline data between the control group and PD group (*n*, %).
**Table S2**: Comparison of serum indicators between the control group and PD group.

## Data Availability

The datasets generated and/or analyzed during the current study are not publicly available due to institutional data governance policies and the need to protect participant confidentiality. However, they are available from the corresponding author on reasonable request, subject to approval by the relevant ethics committee. No other materials were used in this study
