# Supplementary material for: Serum VIP and IL‐1β Levels Are Associated With Anxiety and Depression in Patients With Parkinson's Disease: A Cross‐Sectional Study
Source: Brain Behav. 2026 Sep 1;16(9):e71742. doi: 10.1002/brb3.71742 (PMC13531355; doi:10.1002/brb3.71742)
Supplement: Supplementary file 1 [file BRB3-16-e71742-s001.docx]

Supplementary Material

# Supplementary Tables

| **Item** | **Control group**  **(n=40)** | **PD group (n=56)** | ***t*/*χ^2^*** | ***p*** |
| --- | --- | --- | --- | --- |
| Male [n,(%)] | 21.00（52.50） | 27.00（48.21） | 0.187 | 0.665 |
| Age (years,$\overline{x}\pm s$) | 63.34±5.91 | 62.23±5.37 | 0.852^a^ | 0.397 |
| Hypertension [n,(%)] | 22.00（55.00） | 30.00（53.57） | 0.040 | 0.952 |
| No or low education history [n,(%)] | 8.00（20.00） | 21.00（35.71） | 2.893 | 0.084 |
| Course of disease | - | 3.00（1.00，6.00） | - | - |

**Supplementary Table S1.** Comparison of baseline data between the control group and PD group (n,%)

Note: *t*-test (data conforms to normal distribution).

**Supplementary Table S2.** Comparison of serum indicators between the control group and PD group

| **Item** | **Control group (n=40)** | **PD group (n=56)** | ***Z/t*** | ***p*** |
| --- | --- | --- | --- | --- |
| HCY（μmol/L） | 14.47（12.20，18.84） | 14.18（10.58，18.47） | -0.364^b^ | 0.716 |
| hsCRP（μmol/L） | 1.28（0.80，1.68） | 1.62（0.83，3.71） | -1.498^b^ | 0.134 |
| TC（mmol/L） | 4.13±1.11 | 4.47±1.00 | -1.461^a^ | 0.148 |
| UA（μmol/L） | 298.06±101.55 | 271.20±64.43 | 1.484^a^ | 0.141 |
| VIP（pg/mL） | 219.86±56.82 | 160.37±47.63 | 5.282^a^ | 0.001^**^ |
| TNF-α（pg/mL） | 293.01±41.27 | 371.09±62.84 | -4.836^a^ | 0.001^**^ |
| IL-1β（pg/mL） | 217.10±83.91 | 287.81±116.73 | -3.181^a^ | 0.002^*^ |

Note: a indicates t-test (data follows normal distribution), b indicates Mann-Whitney *U* test (data does not follow normal distribution); **P*<0.05, ***P*<0.001.
